# Frequency of incidental fatty liver on ultrasound and its association with diabetes mellitus and hypertension

**DOI:** 10.12669/pjms.345.15102

**Published:** 2018

**Authors:** Rabbia Zubair, Masoom Mirza, Javeria Iftikhar, Nida Saeed

**Affiliations:** 1 Dr. Rabbia Zubair, FCPS. Assistant Professor. Department of Surgery, Hamdard University Hospital, Karachi, Pakistan; 2 Dr. Masoom Mirza, FRCS. Professor and Head of Surgery Department. Department of Surgery, Hamdard University Hospital, Karachi, Pakistan; 3 Dr. Javeria Iftikhar, FCPS. Senior Registrar. Department of Surgery, Hamdard University Hospital, Karachi, Pakistan; 4 Dr. Nida Saeed, FCPS. Senior Registrar, Department of Endocrine Surgery, Jinnah Post Graduate Medical Centre, Karachi, Pakistan

**Keywords:** Fatty liver, Diabetes mellitus, Hypertension

## Abstract

**Objective:**

To see the frequency of incidental fatty liver on ultrasound and its association with diabetes mellitus and hypertension.

**Methods:**

A cross sectional study was conducted from January 2016 to June 2016 in the department of Surgery and Radiology at Hamdard University Hospital. Patients of both genders and all ages were selected by non purposive convenience sampling. Critically ill, trauma cases and all those patients who had a history of chronic liver disease, alcohol intake and expected pregnancy were excluded. Blood pressure and random blood sugar were recorded.

**Results:**

Six hundred patients were included in the study with mean age of 44.65±18.8 years. 240 patients (40%) had incidental finding of fatty liver on ultrasound. Out of 240 with fatty liver patients 117 were males (48.8%) and 123 were females (51.3%). Total 141 patients were diagnosed with hypertension, out of which 81(57.44%, p< 0.001) had fatty liver on ultrasound. Diabetes mellitus was diagnosed in 84 patients, in which 57 patients (67.8%, p<0.001) had fatty liver.

**Conclusion:**

Fatty liver is a frequently found incidental finding on ultrasound. There is a significant association of fatty liver with diabetes mellitus and hypertension.

## INTRODUCTION

Fatty Liver Disease is rapidly becoming the most common liver disease worldwide. Its prevalence in general population is 20-30% in western countries.^1^ Fatty liver disease is a broad term that has an entire spectrum of liver disease ranging from simple steatosis to nonalcoholic steatohepatitis.^2^ These can lead to liver cirrhosis and hepatocellular carcinoma.^3^ The development of fatty liver involves multiple factors such as dietary habits and sedentary life style.^4^ Fatty liver is a frequent incidental finding on ultrasound abdomen being done for other pathologies. Ultrasonography is the best screening tool to identify fatty liver. Ultrasound compares the echogenicity of liver with renal cortex, both are equal in normal circumstances.^5^ Fatty infiltration of liver may or may not occur with hepatomegaly.^6^

Fatty liver is not only associated with hepatic complications but also extra hepatic complications, like metabolic syndrome, Type 2 Diabetes mellitus (T2DM), and Hypertension (HTN).^7^ Essential hypertension itself is considered an insulin resistant state^8^ and approximately 50% of patients with arterial hypertension are reported to be insulin resistant with hyperinsulinaemia^9^, and the risk of developing essential hypertension increases in the presence of fatty liver. The presence of fatty liver also seems to be involved in the pathogenesis of complications of hypertension and diabetes mellitus, such as Ischemic heart disease, nephropathy, cerebro-vascular events etc.^10^ In fact the most common cause of death in patients with fatty liver is Cardiovascular Disease (CVD).^11^ Therefore, evaluating the associated metabolic disturbances, including T2DM and HTN to reduce CVD in patients with fatty liver is important.^11^

Our study emphasizes on taking incidental fatty liver finding on ultrasound into an account and find its association with newly diagnosed cases of Diabetes mellitus and Hypertension to avoid its disastrous consequences.

## METHODS

This was a cross sectional study carried out at the General surgery and radiology departments of Hamdard University Hospital Karachi, from January 2016-June 2016. Approval from departmental ethical committee has obtained. Total 600 patients who presented in surgical outpatient department were enrolled by non-purposive convenience sampling. Informed consent was obtained from all included patients. Patients of both genders and all age groups were included. Critically ill, trauma cases and all those patients who were having a history of chronic liver disease, viral hepatitis, Wilson’ disease, alcohol intake and expected pregnancy were not enrolled in this study. Already diagnosed cases of Diabetes mellitus Hypertension and those using statins due to Hyperlipidemia were also not included to avoid bias. The diagnosis of diabetes mellitus-DM was made on basis of fasting blood glucose level of ≥126mg/dl and random blood glucose level ≥200 mg/dl^12^ and of hypertension was based on systolic blood pressure ≥140 mm Hg and/or diastolic blood pressure ≥90 mm Hg (measured three times within 30 minutes in the sitting position using a brachial sphygmomanometer. Selected cases had their ultrasound abdomen done to look for fatty liver. Normal echogenicity of the liver is determined by comparing the liver echogenicity with that of the cortex of the kidney. Fatty liver may be diagnosed if liver echogenicity exceeds that of renal cortex and spleen and there is attenuation of the ultrasound wave, loss of definition of the diaphragm, and poor delineation of the intrahepatic architecture. To avoid false-positive interpretations, fatty liver should not be considered present if only one or two of these criteria are fulfilled.^6^

All the information was collected in a specially prepared Performa. The data was entered and analyzed using SPSS version 16.0. The patients were analyzed for age, gender, finding of fatty liver, hypertension and diabetes type 2. Mean was calculated for quantitative variable like age. Frequency (percentage) was calculated for qualitative variables like gender, fatty liver, diabetes mellitus and hypertension. Fischer exact test was used to see, if there existed any statistically significant relation. P-value less than 0.05 was considered significant.

## RESULTS

Six hundred patients were included in this study with mean age of 44.65±18.8 years (Table-I). 46% (n=276) were males and 54% (n=324) were females. Out of all, 240 patients in which 117 males (48.8%) and 123 females (51.3%) had fatty liver. One fifty patients (62.5%) with fatty liver were above the age of 40 years. A total of 141 patients were diagnosed with hypertension, out of which 81(57.44%, p< 0.001) had fatty liver on ultrasound. Diabetes mellitus was diagnosed in 84 patients, in which 57 patients (67.8%, p<0.001) had fatty liver. Forty two (17.5%) patients had no complaints and abdominal pain was found in 108 patients (45%) with fatty liver. Total 204 patients were overweight with BMI 25-29.9, 84 of them had fatty liver (35.0%). 21 patients were obese with BMI >30, 18 of them had fatty liver (7.5%) (Table-II).

**Table-I:** Relation of age with fatty liver.

		Fatty Liver		Total

		Yes	No	
Age Categories	1 - 20 years	9	63	72
		3.8%	17.5%	12.0%
	20 - 40 years	81	102	183
		33.8%	28.3%	30.5%
	40 - 60 years	90	123	213
		37.5%	34.2%	35.5%
	more than 60 years	60	72	132
		25.0%	20.0%	22.0%

Total		240	360	600
		100.0%	100.0%	100.0%

**Table-II:** Relation of BMI with fatty liver.

			Fatty Liver		Total

			Yes	No	
BMI Categories	Less than 18.5	Count	9	30	39
		% within Fatty Liver	3.8%	8.3%	6.5%
	18.5 - 24.99	Count	129	207	336
		% within Fatty Liver	53.8%	57.5%	56.0%
	25 - 29.99	Count	84	120	204
		% within Fatty Liver	35.0%	33.3%	34.0%
	More than 30	Count	18	3	21
		% within Fatty Liver	7.5%	.8%	3.5%

Total	Count	240	360	600
	% within Fatty Liver	100.0%	100.0%	100.0%

## DISCUSSION

Our study examined the frequency of incidental fatty liver on ultrasound and its association with diabetes mellitus and hypertension. In this multiethnic, national, Pakistani hospital based study, we found the frequency of fatty liver to be 40%. The finding of fatty liver is found more in middle age patients, as observed in our study that 62.5% patients with fatty liver were above the age of 40 years. Moreover, our study showed almost equal number of fatty liver in both genders.

In a similar study conducted by Afzal et al, at Sheikh Zayad Hospital Lahore, 130 diagnosed cases of DM-II were evaluated for fatty liver. Out of 130 cases, there were 81 (62%) females and 49 (38%) males. The mean age of the patients was 52.31±5.96. The 61% females were found to have nonalcoholic fatty liver disease, whereas, the 53% males were found to have nonalcoholic fatty liver disease on ultrasound.^12^

A study done at Spain by Lopez-Suaraez A, et al reported the prevalence of fatty liver was 38.5% in the entire sample and 49.5% in hypertensive participants, where as in our study the percentage of fatty liver is 57.4%(p< 0.001). Lopez-Saurez et al; stated the percentage of cases with hypertension was 21.2% greater in individuals with fatty liver than those without fatty liver. Fatty liver was independently associated with prevalent hypertension with an adjusted odds ratio of 1.71 (95% CI, 1.10-2.65, P=0.017). Among non-hypertensive participants, Fatty liver was also independently associated with high-normal systolic blood pressure but not with high-normal diastolic blood pressure.^13^ Rayo et al; also observed clinical association between non-alcoholic fatty liver disease and the development of hypertension.^14^ It stated that development of hypertension is more potentially associated with the more progressive fatty liver than normal or milder state. The incidence of hypertension increased according to the degree of NAFLD (normal: 14.4%, mild: 21.8%, moderate to severe: 30.1%, P<0.001).^14^ Another study conducted by Donati et al., (2003) stated that Hypertensive patients showed a significantly higher prevalence of fatty liver (17/55 patients, 30.9%) than control subjects (7/55, 12.7%; p = 0.041).^15^

Ijaz et al; conducted a study at Nishtar Hospital Multan and reported the frequency of 51% of fatty liver in diabetic patients.^16^ Significant number of diabetic patients in our study had fatty liver (67.8%, p<0.001).

Demographically, it was clear that the mean age of the person to develop NAFLD was greater than 40 years. The mean age to be evaluated in our study was 44.65±18.8 years comparable to the study conducted by Ijaz et al., (2009)^16^ and Luxmi et al., (2008).^17^ We reported 40% cases of fatty liver disease, which was comparable, as per incidence in our neighboring country India, where the frequency was 49% (Amarapurkar et al., 2007).^18^ Whereas, a high prevalence (58.5%) of fatty liver was noted in a study by Afzal et al., (2016). The prevalence 55% was observed in patient of the Arab Peninsula (Akbar and Kawther, 2003).^19^ In another study conducted Targher et al., (2007) reported 69.5% prevalence of fatty liver. Further, it also supported that the prevalence of NAFLD increases with age.^20^ We observed that NAFLD is more or less equally found in women (51.3%) and men (48.8%). Other different studies have been evaluated that the female gender was at more risk for developing NALFD as indicated by Akbar and Kawther (2003).^19^ Ijaz et al; also reported that 62.75% diabetic females develop fatty liver as compared to 37.25%.^16^ In a study conducted by Williams et al., (2011) described that the male patients had higher rates of both NAFLD and NASH as compared to female counterparts. They also reported that diabetic patients are at strong risk for both NAFLD and NASH and this prevalence are increasing with time.^21^ Targher et al., (2005) reported in their study that NAFLD is highly associated with a moderate cardiovascular disease increased risk among type-II risk among diabetic patients.^22^

NAFLD is a silent disease. The majority of individuals with NAFLD have no symptoms and a normal clinical examination.^23^ Children may exhibit symptoms such as abdominal pain, which may be central or in the right hypochondrium, and sometimes fatigue.^24^ In our study, 108(45%) patients with NAFLD presented with abdominal pain, 33(13.8%) presented with vomiting and 42(17.5%) patients had no complaints.

**Fig.1:**
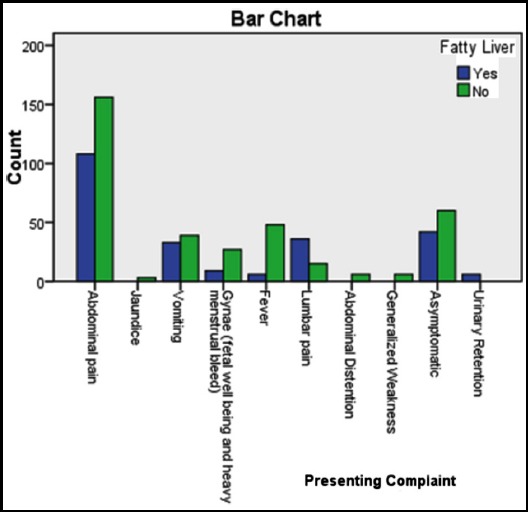
Presenting complains in fatty liver patients.

## CONCLUSION

Fatty liver is a frequently found incidental finding on ultrasound. There is a significant association of fatty liver with diabetes mellitus, hypertension and advancing age. Therefore, it is essential to evaluate those individuals who had an incidental finding of fatty liver on ultrasound. Serum fasting insulin level, fasting blood sugar, fasting lipid profile and liver function tests must be done to assess severity and to avoid disastrous consequences. Liver biopsy can be done where suspicion is high and labs are not supporting the ultrasound findings.

### Authors’ Contribution

**RZ:** Principal Investigator, compilation of data, manuscript writing.

**MM:** Proposed the idea, framed and supervised the research work.

**JI:** Statistical analysis and edited the final manuscript.

**NS:** Data collection and literature search.
